# Effective medium theory to the description of plasmonic resonances: Role of Au and Ti nanoparticles embedded in MoO_3_ thin films

**DOI:** 10.1038/s41598-020-62706-4

**Published:** 2020-04-03

**Authors:** Gesuri Morales-Luna, Michael Morales-Luna

**Affiliations:** 1 Escuela de Ingeniería y Ciencias, Tecnológico de Monterrey, Ave. Eugenio Garza Sada 2501, Monterrey, Nuevo León 64849 México; 2 Escuela de Artes, Arquitectura y Diseño, Universidad de Monterrey, Av. Ignacio Morones Prieto 4500 Pte., A.P, 66238 San Pedro Garza García, Nuevo León México

**Keywords:** Materials science, Materials for devices, Materials for energy and catalysis

## Abstract

The growing interest in functional transition metal oxides for efficient energy consumption or in the bio-sensing process; indicates that is necessary to develop a new theoretical method that describes experiments. This article presents a new theoretical methodology to characterize molybdenum trioxide (MoO_3_) thin films doped with resonant gold – nanoparticles (Au – NPs) and non-resonant titanium – nanoparticles (Ti – NPs). The modulation of surface plasmon resonance (SPR) and the implications in the MoO_3_ transmittance spectrum is described by applying an effective medium theory. The transmittance modulation was modified by variating three parameters, the radius of the NPs, the concentration of the NPs as well as the variation of the MoO_3_ thin films thickness. It was found that the nanoparticles concentration is the most important parameter in the transmittance modulation. Additionally, the orthorhombic and monoclinic structure of MoO_3_ was studied, from which it was obtained that the monoclinic structure of the MoO_3_ doped with Au – NPs favors the reduction in the transmittance values in the visible region which is associated with the increase of the SPR signal. Similar analyses are performed for non-resonant nanoparticles such as Ti, where it was found that optical modulation is not as marked as the case of gold nanoparticles.

## Introduction

Optical properties of different kind of systems has attracted attention for many researchers around the world^1–5^. Developing new devices with interesting optical properties has been a technological and experimental challenge^6,7^. In order to describe optical properties from a theoretical point of view, it have been developed sophisticated multiphysics modeling finite element commercial software, such as Comsol Multiphysics, Lumerical FDTD or BEM which allow you to calculate, reflection or transmission frequency-dependent or scattering parameters and near or far electromagnetic field projections. However, many of them are difficult to access since they are expensive or they are not very intuitive software’s. So, implementing new theoretical formalisms to characterized and analyzed thin films with new, interesting and unexpected effects could be useful for developing new devices with different kinds of optical properties. There are some different approaches to characterize thin films^8–10^, particularly for thin films doped with different materials, using an effective medium theory to describe the radiation interaction with the doped thin films could be an alternative. In effective medium models, one of the most important approximation is the large size limit. The most studied formalisms that consider effective medium theories (EMTs) are Maxwell Garnett or Bruggeman, these models are applied to described materials with granular topology^11^ and materials with intermixed components^12^, respectively. These EMTs are based on small spheres approximation, which means that the size of the particles is small compared with the incident radiation wavelength^13^. An alternative formalism to study effective mediums is the refractive index proposed by van de Hulst^14,15^. This simple theoretical approach has been used at different configurations; to mentioned some, as colloidal systems^15–17^, or colloidal monolayers^10^, obtaining good agreement with experimental measurements. The main difference with Maxwell Garnett and Bruggeman is that van de Hulst depends on the forward scattering amplitude, which predominantly depends on the nanoparticle size. Hence, this approach can be more robust since not have the size particle limitation, that is the case of the two EMTs, as already mentioned. Being this, one important factor in device design for specific applications. Combining the effective medium approach with well stablished reflection or transmission coefficients^18^ to predict the behavior of undoped and doped thin films, could be a novelty method to study thin films.

Employing this model to describe the behavior of various materials specifically the transition metal oxides (TMOs) such as molybdenum trioxide (MoO_3_), tungsten trioxide (WO_3_) and titanium dioxide (TiO_2_), to name a few of them, will be useful to predict the material optical behavior^19–21^. In recent years, MoO_3_ has been extensively studied for their potential applications as a biosensing, temperature or gases sensors, also, as a layer in solar cell devices, photocatalytic material or as an electrochromic layer in smart windows for efficient energy consumption applications in buildings^7,19,21–24^^,^. Different deposition techniques have been studied such as sputtering rf or dc, sol-gel and evaporation, in order to improve their properties for different applications^19,24^. In addition to deposit techniques, different configurations of TMOs such as MoO_3_ doped with ZnSe nanoparticles, W_1–*x–y*_Ti_*x*_Ni_*y*_O_3_, W_1*–x–y*_Ti_*x*_Mo_*y*_O_3_, or as two-layer systems MoO_3_ / CdSe, Pt / MoO_3_ and Au / MoO_3_ have been studied^24–28^. However, an interesting phenomenon arises when doping some of the TMOs with some kind of nanoparticles, *e.g*., doped MoO_3_ with Au nanoparticles^29^. The metallic nanoparticles (NPs) incorporation in the molybdenum oxide matrix extremely modifies the optical properties; mainly associated with surface plasmon resonance (SPR)^30^. The phenomenon arises from the interaction of the electromagnetic field of an incident light beam on the surface of the material that derives in collective oscillations of free-charge carriers^31^. This phenomenon radically improves the optical modulation by providing unprecedented control over light-induced excitation which is a priority in the chromogenic properties where it wants to be used the photogenerated electrons originated by visible light irradiations. Even more, these same photogenerated electrons can be used in photocatalytic experiments where it is desirable to absorbed visible light for the degradation of inorganic compounds in aqueous systems^24,32^.

From the above, and from some reports mention that the MoO_3_ could serve as a material that traps electrons originated by the SPR phenomena due to its features as wide bandgap ~ 3 eV, as well as the external valence electrons in the shell *d* of molybdenum which are ideal for SPR absorption^29,33^. Thus, MoO_3_ becomes an interesting material to study the optical changes caused by SPR phenomena. Nonetheless, MoO_3_ shows mainly two different structures. The orthorhombic or *α* – phase that is thermodynamically stable, and consist of MoO_6_ octahedra share corners, where the oxygen anions, O, are located at the vertices of each octahedron and the Mo cation is located at the center. In this structure the octahedra share edges and form a two-dimensional layered structure^34^. Additionally, the monoclinic structure or metastable *β* – phase has been reported. This structure shows the same octahedra formation as the *α* – phase. However, octahedra are connected by vertices^33^. Several authors have found that the *α* – MoO_3_ shows a lower response than the *β* – MoO_3_ for different applications, *e.g*., the chromogenic response^19,33^. This result has been explained in terms of tunnel structural formation in *β* – MoO_3_, a place where intercalated ions can be used as a mobility place that is a crucial element in the electrochromic mechanism^35^.

Thus, the effect of the *α* – MoO_3_ and *β* – MoO_3_ on the modulation of surface plasmon resonance will be analyzed, as well as the variation of the MoO_3_ thin films thicknesses. Subsequently, will be studied the effect of the transmittance spectra (evolution of the SPR phenomenon) of MoO_3_ thin films doped with Au – NPs. It should be noted that these nanoparticles are considers as a noble metal, known as plasmonic particles^36^. In order to corroborate the plasmonic effects, it will be analyzed the case of MoO_3_ thin films doped with Ti – NPs, which corresponds to a non-resonant particle^37^. As a complement, the concentration, and NPs radius will be studied. These studies will be carried out by the implementation of the effective medium theory that opens a novelty alternative for the optical characterization of different thin films particularly in this work it will be focused in MoO_3_ thin films. Specifically, to our knowledge, in the literature, the van de Hulst model has not been applied to describe the optical modulation caused by nanoparticles embedded in a thin film. Previously van de Hulst model was used to described colloidal systems. Thus, in this work, we propose a simple EMT that could be used to describe the optical modulation of doped MoO_3_ thin films with Au – and/or Ti – NPs which are of interest in areas of energy efficiency in buildings, in clean energy generation applications or even more bio-sensing process^19,25^.

## Theoretical Background

The theoretical modeling of the undoped and doped MoO_3_ thin films is carried out through the effective media theory that considers certain particles immersed in a matrix medium that could be considered as a dilute turbid system where the volume occupied by the particles is small compared to the total volume, the effective refractive index is modeled with the van de Hulst approximation. Additionally, is well known that the particle’s contribution depends on the refractive index, shape and particles’ size^38^. Considering this, the effective refractive index is given by^15,16^,

1 $${n}_{vdH}={n}_{m}\left[1+\frac{3\,i\,f}{2\,{x}^{3}}S(0)\right],$$

**Figure 1:**
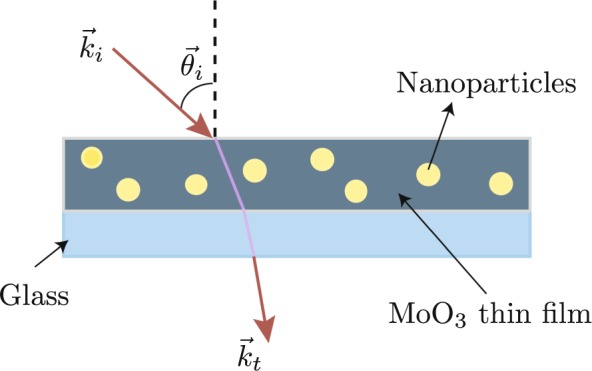
Schematic of nanoparticles-doped MoO_3_ thin films system, deposited on a glass substrate.

${n}_{m}$ is the refractive index of the matrix, where the particles are immersed, $f$ is the volume filling fraction, $x$ is the size parameter given by $x={k}_{o}{n}_{m}a$, where ${k}_{o}=2\,\pi /\lambda$ is the wavenumber in vacuum, $a$ is the radius of the particles and $\lambda$ is the wavelength, $S(0)$ is called the forward scattering amplitude given by the expression $S(0)=\sum _{n=1}[(2n+1)/2]({a}_{n}+{b}_{n})$ where *a*_*n*_ and *b*_*n*_, are known as scattering coefficients, they are complex functions of Ricatti-Bessel and its derivatives, more details are reported in ref. ^39^. It is known that the contribution of this parameter will be more evident for cases of particles with radius bigger than 10 nm^39^. To infer the optical properties of doped thin film, it is necessary to consider the reflection or the transmission coefficients of the system as can see in Fig. 1. In this work, the transmittance will be used as the tool to characterized thin films. The system diagram to study is a three-layer system as shown in Fig. 1.

For the proposed analysis in this work, the transmission coefficient that is going to be used is the well-known composition equation that relates the transmission amplitude for the interface between media 1 and 3 as well as the transmission amplitudes between media 1–2 and media 2–3^17^ which is given by

2 $${t}_{123}^{j}=\frac{{t}_{12}^{j}{t}_{23}^{j}{e}^{i{k}_{z}^{eff}h}}{1+{t}_{12}^{j}{t}_{23}^{j}{e}^{2i{k}_{z}^{eff}h}},$$

the super index $j$ is associated to the two possible polarizations *s* or *p* which depends on the case that is working on. Finally, the *h* parameter is the thin film thickness. For the *s* polarization the terms of Eq. (2) can be rewrite for the first interface as:

3 $${t}_{12}^{s}=\frac{2\,{k}_{1z}}{{k}_{1z}+{k}_{2z}},$$

on the other side, for *p* polarization

4 $${t}_{12}^{p}=\frac{2{n}_{2}{n}_{1}{k}_{1z}}{({n}_{1}^{2}{k}_{2z}+{n}_{2}^{2}{k}_{1z})},$$

where ${n}_{1}$ and ${n}_{2}$ are the refractive index of media 1 and 2, ${k}_{lz}$ are the wavenumbers in each media, given by ${k}_{1z}={k}_{o}{n}_{1}\,\cos \,{\theta }_{i}$ and ${k}_{2z}={k}_{o}\sqrt{{n}_{2}^{2}-{n}_{1}^{2}{\sin }^{2}{\theta }_{i}}$, here ${\theta }_{i}$ is the incidence angle. Now, for the second interface, media 2 and 3, the transmission coefficient for *s* polarization is given by,

5 $${t}_{23}^{s}=\frac{2{k}_{z}^{eff}}{{k}_{z}^{eff}+{k}_{3z}},$$

and for *p* polarization is

6 $${t}_{12}^{p}=\frac{2{n}_{3}{n}_{vdH}{k}_{z}^{eff}}{({n}_{vdH}^{2}{k}_{3z}+{n}_{3}^{2}{k}_{z}^{eff})},$$

here ${n}_{vdH}$ is the refractive index given by Eq. (1), and ${n}_{3}$ is the refractive index of medium 3. For the wavenumber in medium 3, *k*_3z_ is given by ${k}_{o}\sqrt{{n}_{3}^{2}-{n}_{1}^{2}{\sin }^{2}\,{\theta }_{i}}$ and for the effective wavenumber

7 $${k}_{z}^{eff}={k}_{o}\sqrt{{n}_{2}^{2}-{n}_{1}^{2}{\sin }^{2}{\theta }_{i}+2{n}_{2}^{2}\left[\frac{3i\,f}{2{({k}_{o}{n}_{2}a)}^{3}}S(0)\right]}.$$

Lastly, the transmittance can be defined as,

8 $${T}_{123}^{j}={|{t}_{123}^{j}|}^{2}\left[\frac{{\rm{Re}}({k}_{3z})}{{k}_{1z}}\right]$$

As can be seen from this last relation, the dependence falls on well-known parameters of the materials such as the refractive index and the filling fraction. From here can be considered as the concentration of NPs immersed in the MoO_3_ matrix. So, different kinds of thin films can be considered. Also, different nanoparticles can be used to doped the thin films. This work will show interesting cases for different applications as higher electron harvesting or optical modulations, to mentions some of them^22,24–26^. Therefore, it will be focus on the two phases of MoO_3_, as already mentioned. Additionally, will be studied the effect of Au – and Ti – NPs doped MoO_3_ thin film. To compute the theoretical analysis, refractive indexes values of MoO_3_, Au and Ti, were obtained from refs. ^40–42^.

## Analysis and Results

### Numerical results

This section will be presented the transmittance spectrum as a function of wavelength in range from 300–1800 nm for each analysis. Different parameters like NPs radius, NPs concentration and thickness will be modified, ranging between 5–15 nm, 0–4% and 80–130 nm, respectively. Two different kinds of refractive index of MoO_3_ will be considered, one for the called $\alpha -{{\rm{MoO}}}_{3}$ and other for the $\beta -{{\rm{MoO}}}_{3}$^40^ both structures will be studied doping with gold and titanium nanoparticles.

### ${\boldsymbol{\alpha }}{\boldsymbol{-}}{\bf{M}}{\bf{o}}{{\bf{O}}}_{{\bf{3}}}$ and ${\boldsymbol{\beta }}{\boldsymbol{-}}{\bf{M}}{\bf{o}}{{\bf{O}}}_{{\bf{3}}}$ thin films

**Figure 2:**
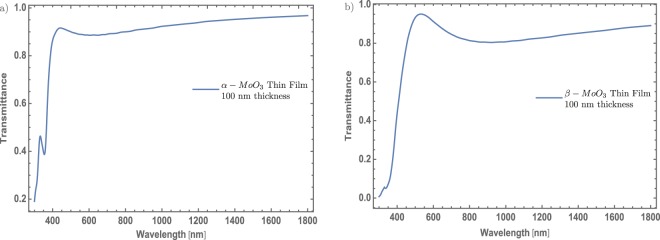
(**a**) Typical $\alpha -{{\rm{MoO}}}_{3}$ thin film transmittance spectrum as function of wavelength. (**b**) Typical $\beta -{{\rm{MoO}}}_{3}$ thin film transmittance spectrum as function of wavelength. In both spectra, the thickness was fixed at 100 nm.

The transmittance spectra of $\alpha -{{\rm{MoO}}}_{3}$ and $\beta -{{\rm{MoO}}}_{3}$ thin films are display in Fig. 2(a,b), respectively. These spectra are similar to transmittance spectra reported by Kumar *et al*.^29^ and corroborate the good agreement with the proposed theoretical model. Just to take into account, to simulate these spectra it was consider a normal incidence of the electromagnetic wave where the *s* or *p* polarization will be the same. Figure 2(a) displays the *α* – MoO_3_ thin films transmittance spectrum with a thickness fixed value of 100 nm. From this figure, it can be seen the fundamental transmittance edge around 420 nm which is similar to the experimental measurements^29^. Additionally, this result tells that the MoO_3_ thin film stands out as a transparent material at least for the visible and near-infrared (NIR) spectrum. On the other hand, the transmittance spectrum of $\beta -{{\rm{MoO}}}_{3}$ is shown in Fig. 2(b). This figure shows a similar behavior as Fig. 2(a); however, the transmittance decreases its value around 10% comparing with $\alpha -{{\rm{MoO}}}_{3}$ thin film. For specific applications where is desirable to modulate some part of the electromagnetic radiation, *e.g*., around the visible range, the use of MoO_3_ alone will not be enough to modulate the visible electromagnetic radiation. This is the reason why doped MoO_3_ thin film can be interesting to discuss. Hence, it will be investigated the evolution of the MoO_3_ thin films transmittance spectra with different (Au and Ti) nanoparticles concentrations. Additionally, to confirm that our results are compatible with the description of the MoO_3_ thin films, the value of the band gap was computed by the Tauc’s model that considers an indirect bandgap transition. The obtained values are between 3.13 and 3.15 eV for the α – phase and between 2.97 and 3.00 eV for the β – phase, these values are similar to those reported previously^19,25,26^.

### ${\boldsymbol{\alpha }}{\boldsymbol{-}}{\bf{M}}{\bf{o}}{{\bf{O}}}_{{\bf{3}}}$ and ${\boldsymbol{\beta }}{\boldsymbol{-}}{\bf{M}}{\bf{o}}{{\bf{O}}}_{{\bf{3}}}$ thin films doped with Au nanoparticles

**Figure 3:**
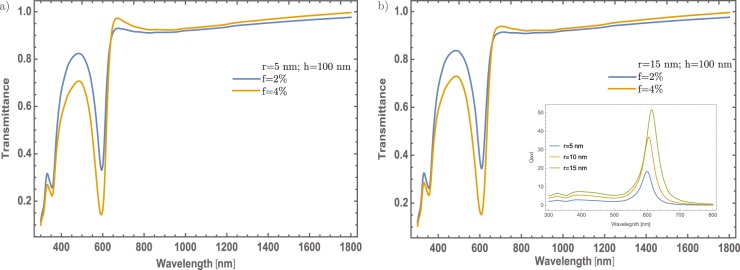
(**a**) Transmittance spectra of $\alpha -{{\rm{MoO}}}_{3}$ thin film. It was fixed the thickness and the Au – NPs radius values at 100 nm and 5 nm, respectively. (**b**) Transmittance spectra of $\alpha -{{\rm{MoO}}}_{3}$ thin film. It was fixed the thickness and the Au – NPs radius values at 100 nm and 15 nm, respectively. Blue and orange lines correspond for 2% and 4% concentrations, respectively in both figures. The extinction efficiency coefficient spectra of gold NPs immersed in $\alpha -{{\rm{MoO}}}_{3}$ is shown as an inset for three different radii, 5, 10 and 15 nm, showing a small red-shifting as the radius increase.

In Fig. 3, a significant change in the transmittance spectrum of MoO_3_ thin films can be observed, mainly in the visible region. The generated band (between 500 and 700 nm) with a minimum value around 600 nm is associated to the surface plasmon resonance due to the incorporation of Au – NP in the MoO_3_ matrix, that might corroborate the validation of the proposed theoretical model by the experimental observation presented in reference 29. From these results, it is interesting to study different concentrations of Au in the MoO_3_ matrix, for possible applications where the transmittance modulation in the visible range, could be the most important parameter^23–25^. The proposed model considers three important parameters in the thin films doping: the radius of the NPs, the volume filling fraction (also called as the particle concentration) and the thickness of the thin films. Firstly, it will be discussed the effect of the NPs radius (5 and 15 nm) in the transmittance spectrum of the MoO_3_ for $\alpha$ – phase. Two parameters were fixed, the Au concentrations (2 and 4%) and the thin films thickness at 100 nm, see Fig. 3(a,b), respectively. Figure 3(a) show the transmittance spectra considering a radius of 5 nm for the Au – NPs at two different concentrations. For the concentration of 2% (blue line), it can be observed the feature band associated with the SPR signal centered at wavelength of λ = 600 nm. This implies, a decrease around of 0.6 on transmittance with respect to the thin film without NPs. By increasing the Au – NPs concentration at 4% (orange line), it can be seen that this change is even better around 0.75 on transmittance. Thus; if the concentration increases, the transmittance has a decrease in the visible range. On the other hand, by increasing the radius of the nanoparticles around 15 nm, see Fig. 3(b), there is no significant difference as compared with Fig. 3(a). This shows that the most important parameter, that modifies the MoO_3_ thin films transmittance, is the NPs concentration change.

**Figure 4:**
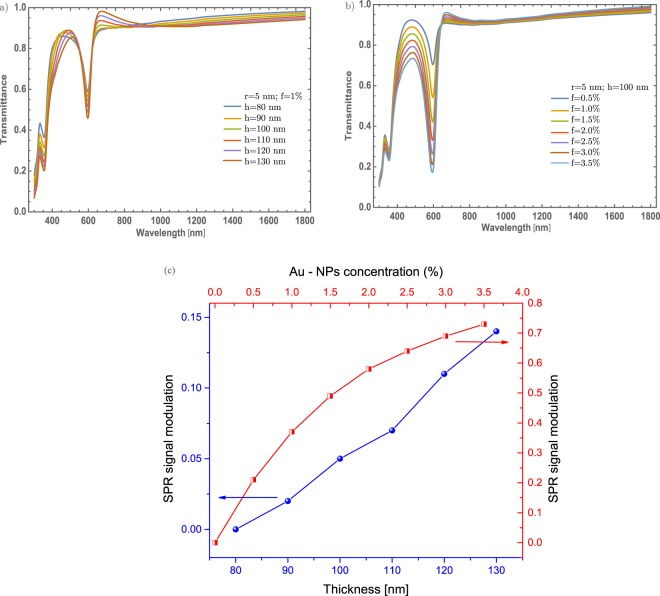
(**a**) Transmittance spectra of $\alpha -{{\rm{MoO}}}_{3}$ thin film with different thin film thickness from 80 nm to 130 nm. The Au – NPs radius and the Au concentration were fixed at 5 nm and 1%. (**b**) Transmittance spectra of $\alpha -{{\rm{MoO}}}_{3}$ thin film with different concentrations from 0.5% to 3.5%, the respective labels are shown in this figure. The Au – NPs and thickness values were fixed at 5 nm and 100 nm, respectively in this figure. (**c**) SPR signal modulation as a function of a thickness (blue spheres, left-hand and bottom scale) and SPR signal modulation as a function of Au – NPs concentration (red half-squares, right-hand and top scale). The arrows indicate the corresponding axes.

As already shown, the NPs radius not contributes significantly in the optical modulation in this particular case. As it is known while the radius of the nanoparticle increases a red-shifting on the extinction efficiency spectra is presented^43^, as can see in the inset of the Fig. 3(b). As it can see, there are not significant red-shift changes. As will be described in section Mechanism to enhanced the optical properties, there are no significant changes associated with the SPR signal that could be associated with a higher crystallinity domain which is a feature of the *α* – phase. So, in the following results it was fixed at 5 nm the radius of the Au – NPs. Thus, studying different cases (thickness and concentrations) could give more physical insight to understand better the optical properties of these doped thin films. In Fig. 4 are presented two different situations. On the one hand, Fig. 4(a) the concentrations of Au – nanoparticles are fixed at 1%, and the thickness was varied from 80 nm to 130 nm, in steps of 10 nm. As was mentioned, one purpose is to tune the transmittance at the visible range albeit there is a little modulation of the resonance being bigger the dip around 600 nm, due to the thickness of the thin film. The changes are not significant as the thin film thickness increases. On the other hand, Fig. 4(b) displays the evolution of the SPR signal as varying the concentration of Au – NPs; in which, around of 0.55 on transmittance will be changed in visible range (~600 nm) if it is compare with the 0.5% and 3.5% concentration. Also, there is a decreasing of 0.35 on transmittance around 450 nm to 550 nm, giving in general, a reduction of transmittance around the visible range. To be more clearly with the description of the SPR signal, it was analyzed the signal as a function of the thickness and the Au nanoparticles concentration, see Fig. 4(c). As can be seen, the increase in the SPR signal has a linear behavior; by increasing the thickness. On the other hand, as the concentration of nanoparticles increases, the SPR signal grows faster compared to the change in thickness and it is clear that the evolution of the SPR signal is similar to a logarithmic function. The behavior of the Au – NPs as doped material, in this configuration, gives an important modulation of the optical properties.

**Figure 5:**
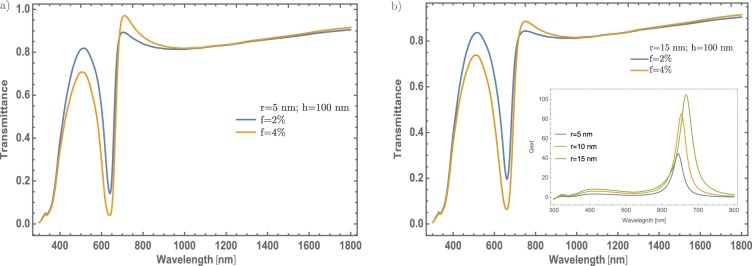
(**a**) Transmittance spectra of $\beta -{{\rm{MoO}}}_{3}$ thin film for two different concentrations (2 and 4%). The thickness and Au – NPs radius are fixed at 100 nm and 5 nm, respectively. (**b**) Transmittance spectra of $\beta -{{\rm{MoO}}}_{3}$ thin film for two different concentrations (2 and 4%). The thickness and Au – NPs radius are fixed at 100 nm and 15 nm, respectively. Blue and orange lines for 2% and 4% concentration, respectively they are labeled in both figures. The extinction efficiency spectra of gold NPs immersed in $\beta -{{\rm{MoO}}}_{3}$ is shown as inset for three different radii, 5, 10 and 15 nm, showing a big red-shifting as the radius increase.

As a comparative now considering the case of ${{\rm{MoO}}}_{3}$ thin film with $\beta -$ phase using the same parameters as the $\alpha -{{\rm{MoO}}}_{3}$. Figure 5 depicted the transmittance spectra of $\beta -{{\rm{MoO}}}_{3}$. As Fig. 5(a,b) shows, they are similar as compare with Fig. 3(a,b). However, the particle size contribution in this case is more significant due to that red-shift that is observed in the transmittance dip which is more marked than the *α* – phase, also seen in the extinction efficiency coefficient plotted as an inset in Fig. 5(b). In this case, the MoO_3_ shows an amorphous structure that might be related to a higher free electron density. More details of this behavior will be discussed in section Mechanism to enhanced optical properties. Other difference is the band centered at 350 nm vanish in $\beta -{{\rm{MoO}}}_{3}$, this phenomenon is due to the refractive index of the ${{\rm{MoO}}}_{3}$ in this phase, which means that the structure of this material is quite different compared with $\alpha$ – phase. It should be noticed that for the orthorhombic structure of MoO_3_, the radius of the nanoparticles, is not a strong parameter for tuning the intensity of the SPR signal.

**Figure 6:**
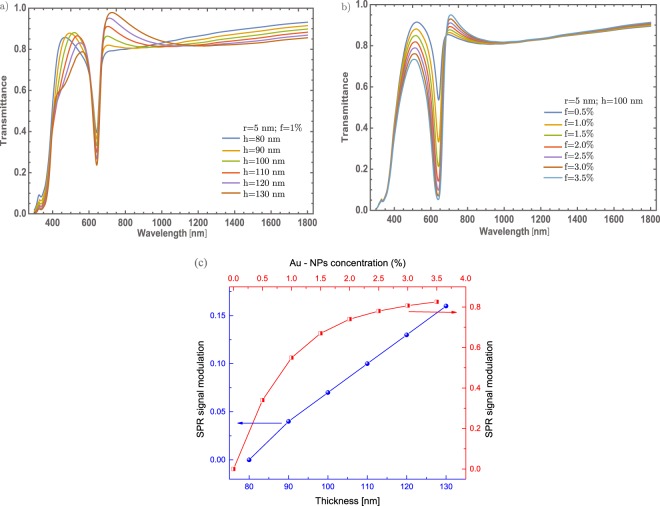
(**a**) Evolution of the transmittance spectra of $\beta -{{\rm{MoO}}}_{3}$ thin film at different thickness from 80 nm to 130 nm. The Au – NPs radius and Au concentration was set at 5 nm and 1%, respectively. b) Evolution of the transmittance spectra of $\beta -{{\rm{MoO}}}_{3}$ thin film at different concentrations from 0.5% to 3.5%. The thickness and Au – NPs radius was set at of 100 nm and 5 nm, respectively. The respective labels are shown in the figure. (**c**) SPR signal modulation as a function of a thickness (blue spheres, left-hand and bottom scale) and SPR signal modulation as a function of Au – NPs concentration (red half-squares, right-hand and top scale). The arrows indicate the corresponding axes.

For completeness, the thickness variation of the thin films will be studied, see Fig. 6(a). The transmittance spectrum shows a better modulation of the SPR signal as the thin film thickness is increased. This phenomenon is a little different from the orthorhombic structure, where it was possible to modulate up to 40% for a thickness value of 130 nm. Though, in the case of the monoclinic structure, the transmittance can be modulated up to 70% at the same thickness value. But the transmittance modulation is improved when the concentration of the gold nanoparticles was increased [Fig. 6(b)], same behavior was observed in the $\alpha$ – phase. Nevertheless, for the $\beta$ – phase it was possible to modulate up to 85%, for gold nanoparticles concentration of 3.5%. Also, the resonance decreases a little if is compared with Fig. 4(b), around 600 nm, which indicate that the transmittance decreases around 5% at this wavelength. The simulated transmittance response in this $\beta$ – phase doped with Au – NPs, giving a reduction in the visible region, but also gets a decrease in the IR range. Similar to the study conducted for the *α* – phase, it was analyzed the SPR signal as a function of the thickness and the Au nanoparticles concentration, as can be seen in Fig. 6(c). The SPR signal shows again a linear behavior; by increasing the thickness. Nevertheless, as the concentration of Au – NPs increases, the SPR signal grows faster compared to the change in thickness and even more if its compare with the behavior of the alpha phase [Fig. 4(b)]. It is clear that the evolution of the SPR signal is similar to a logarithmic function and it shows the best SPR modulation.

**Figure 7:**
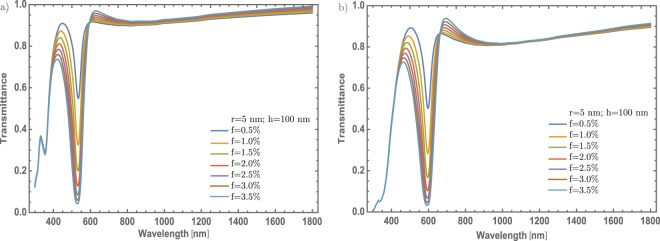
(**a**) Transmittance spectra of $\alpha -{{\rm{MoO}}}_{3}$ thin film at seven different concentrations from 0.5% to 3.5%. b) Transmittance spectra of $\beta -{{\rm{MoO}}}_{3}$ thin film at different concentrations from 0.5% to 3.5%, The thickness and the Ag – NPs radius was set at 100 nm and 5 nm, respectively. The respective labels are shown in the figure.

Something that we should mentioned is that another configuration was analyzed, but with few examples, due to the similar behavior with Au – NPs. This configuration is $\alpha -{{\rm{MoO}}}_{3}\mbox{--}{\rm{Ag}}$ (Silver) and $\beta -{{\rm{MoO}}}_{3}-{\rm{Ag}}$, varying the concentration for a radius and thickness thin films fixed, see Fig. 7. This configuration shows same behaviors as the configuration previously studied, but with some differences. The most important difference is the blue-shift of the resonance that is around 550 nm, compared with the Au cases that is at 600 nm [see Fig. 4(b)], something expected due to the behavior of the Ag – and Au – NPs. The blue-shift at Fig. 7(b) is smaller compared with Fig. 6(b), comparing the values, for the silver case the resonance is centered at 600 nm and for the gold case is centered at 650 nm, which is important to mention that the phase in the MoO_3_ have an important role in the behavior of modulation resonance. Additionally, at this wavelength the transmittance decreases 0.1 on transmittance value for the concentration of 3.5%, as depicted in Fig. 7(a). For the case of $\beta -{{\rm{MoO}}}_{3}-{\rm{Ag}}$, Fig. 7(b) shows the transmittance spectra for different concentrations, the SPR signal shows a red-shift compared with the resonance depicted in Fig. 7(a). This is the principal contribution of the $\beta$ – phase and the Ag – NPs in the transmittance spectra. The resonance has a similar behavior compared with the $\alpha$ – phase, but the dip decreases little more than the $\alpha$ – phase in the Au – NPs case. Hence, these results indicate the predominant role of Ag – NPs concentration in SPR modulation, over the NPs and the thickness of the thin films.

### ${\boldsymbol{\alpha }}{\boldsymbol{-}}{\bf{M}}{\bf{o}}{{\bf{O}}}_{{\bf{3}}}{\boldsymbol{-}}{\bf{T}}{\bf{i}}$ and ${\boldsymbol{\beta }}{\boldsymbol{-}}{\bf{M}}{\bf{o}}{{\bf{O}}}_{{\bf{3}}}{\boldsymbol{-}}{\bf{T}}{\bf{i}}$ thin films

**Figure 8:**
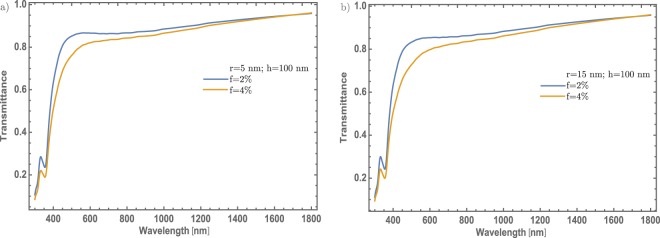
(**a**) Transmittance spectra of $\alpha -{{\rm{M}}{\rm{o}}{\rm{O}}}_{3}$ thin film at two different concentrations (2 and 4%). It was fixed the Ti – NPs and thickness at 5 nm and 100 nm, respectively. (**b**) Transmittance spectra of $\alpha -{{\rm{MoO}}}_{3}$ thin film at two different concentrations (2 and 4%). It was fixed the Ti – NPs and thickness at 5 nm and 100 nm, respectively. Blue line for 2% and orange line for 4% concentration, are label for both figures.

Finally, it will be tested the theoretical model with another material commonly used as a dopant in the MoO_3_ thin films, for different applications as the titanium^27,28^. The used parameters in the previous sections will be the same in this case, only with a variation of thicknesses, which are bigger in these cases, with the aim to compare and take a look at which configuration could be more useful for the optical modulation. Figure 8(a,b) depicted the $\alpha -{{\rm{MoO}}}_{3}$ transmittance spectra for different Ti – NPs, 5 and 15 nm, respectively with a fixed concentration at 2 and 4% and thickness value of 100 nm. It is worth mention here, that firstly the $\alpha -{{\rm{MoO}}}_{3}$ will be analyzed. As was previously discussed and corroborating what was found in the previous study; the size of the NPs is not a relevant parameter in the optical modulation. Nevertheless, if it compared these results with the Au – NPs there is no apparent SPR signal. This could corroborate that this abrupt transmittance decrease is associated only to the Au – NPs. On the other hand, we can justify the incorporation of Ti into the MoO_3_ matrix because the transmittance spectrum shows slight differences as compared with Fig. 2(a).

**Figure 9:**
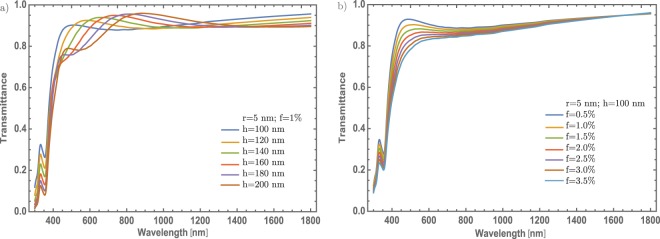
(**a**) Transmittance spectra of $\alpha -{{\rm{MoO}}}_{3}$ thin film at different thickness from 100 to 200 nm doped with Ti – NPs of radius of 5 nm with 1% concentration of Ti. (**b**) Transmittance spectra of $\alpha -{{\rm{MoO}}}_{3}$ thin film at different concentrations from 0.5% to 3.5%, with a thickness value of 100 nm, and 5 nm radius of the Ti – NPs. The respective labels are shown in the figure.

In the same way as the section of thin films doped with Ti nanoparticles, let’s investigate which is the effect of varied the MoO_3_ thin film thickness, fixing the Ti – NP radius at 5 nm and the concentration at 1%. Figure 9(a) displays the transmittance evolution as the thickness increase from 100 to 200 nm. As Liu *et al*^7^. reported TMOs are good candidates to be plasmonic materials which combined with non-resonant particles can be generated a band centered at 450 nm which suffers a red-shift to a value ~ 820 nm and as Liu mentions, this band is associated to the SPR phenomenon. So, the incorporation of Ti – NPs, in the MoO_3_ can increase the SPR signal of MoO_3_ and the red-shift corroborate the presence of the resonant electrons as the thickness increases. Figure 9(b) shows the transmittance evolution as the concentration increases. In this figure the radius and the thickness are fixed at 5 nm and 100 nm, respectively. Here the transmittance modulations increase as the concentration increases. This behavior is slightly similar to those found in section of thin films doped with Ti nanoparticles, nonetheless, the transmittance modulation does not change significantly.

**Figure 10:**
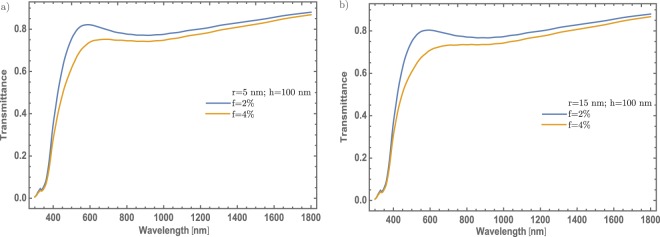
(**a**) Transmittance spectra of $\beta -{{\rm{MoO}}}_{3}$ thin film at two different concentrations (2 and 4%). The thickness and the Ti – NPs were set at 100 nm 5 nm, respectively. (**b**) Transmittance spectra of $\beta -{{\rm{MoO}}}_{3}$ thin film, for two different concentrations (2 and 4%). The thickness and the Ti – NPs were set at 100 and 5 nm, respectively. Blue line for 2% and orange line for 4% concentration, are label for both figures.

Therefore, comparing these results with the Au – NPs doped $\alpha -{{\rm{MoO}}}_{3}$ thin films, the use of Au – NPs embedded in MoO_3_ shows the best transmittance modulation in the visible range. Now it will be considered the change of the MoO_3_ structure. Figure 10(a,b) shows the MoO_3_ transmittance spectra for the NPs radius of 5 and 15 nm, respectively. In this case, the NPs concentration and the thickness were fixed with the same values as in the section of thin films doped with Au nanoparticles. Beside, the transmittance spectrum still shows particular features such as the fundamental transmission edge around 420 nm, a characteristic associated with MoO_3_ thin films.

**Figure 11:**
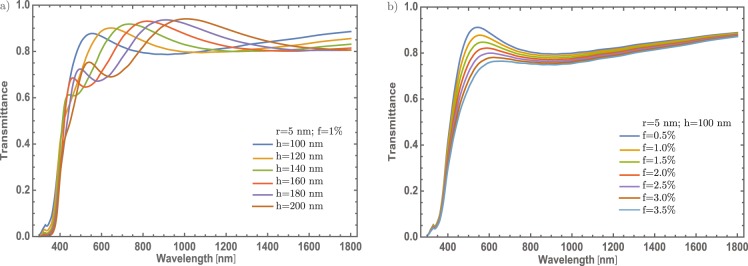
(**a**) Transmittance spectra of $\beta -{{\rm{MoO}}}_{3}$ thin film at different thickness from 100 nm to 200 nm. The Ti – NPs radius and Ti concentration was set at 5 nm and 1%, respectively. (**b**) Transmittance spectra of $\beta -{{\rm{MoO}}}_{3}$ thin film at different concentrations from 0.5% to 3.5%. The thickness and Ti – NPs radius were set at of 100 nm and 5 nm, respectively. The respective labels are shown in the figure.

Finally, if the concentration is fixed at 1% and considering 5 nm of radius for Ti nanoparticles and leaving free the MoO_3_ thin film thickness, the results are shown in Fig. 11(a). Additionally, if the Ti – NPs radius and thickness thin films are fixed at 5 nm and 100 nm, respectively, and considering the concentration as a variable parameter, the transmittance spectra are depicted in Fig. 11(b). However, is interesting to mention that there is a red-shift in the maximum transmittance due to the MoO_3_ from the peak centered at 450 nm to 1000 nm as the thickness is increasing from 100 to 200 nm, respectively, as shown in Fig. 11(a). These results are similar as in Fig. 9(a), which means that the transmittance changes are affected by the thickness of the thin film. But in the case of *β* – phase, the red-shifting of the SPR band is more intense and corroborates that was found for the case of Au – NPs. Thus, for the non-resonant particles, the behavior is the same, showing a remarkable red-shift of the SPR band as can observe in Fig. 11(a) as compared with Fig. 9(a). Additionally, in Fig. 11(b), there is a slight decrease in transmittance at the NIR range as compared to Fig. 9(b). This leads us to conclude that the monoclinic structure of the MoO_3_ also enhances the transmittance modulation, unlike the orthorhombic structure where the transmittance changes are not promising.

By looking Figs. 4 to 7, attractive optical properties for developing new devices were found, the plasmonic effect that is observed on the transmittance spectra could be of interest to researchers. Also, the effect of the Ti – NPs over the MoO_3_ thin film has less impact in the transmittance spectra in the visible range, than the Au – NPs or even in Ag – NPs. These results show the importance of plasmonic nanoparticles embedded in MoO_3_ thin films, opening a new methodology to characterized and developing new functional materials.

### The mechanism to enhance optical properties of MoO_3_ thin films doped with resonant and non-resonant particles

Due to the MoO_3_ thin films have a very wide technological application, understanding the modulation mechanism of the transmittance spectrum when it is doped with plasmonic nanoparticles and non-resonant particles, it becomes a fundamental aspect in the research. Therefore, this section describes the possible mechanism of the differences in the optical modulation that was presented by the *α* – phase and *β* – phase. On the one hand, it was shown that the *β* – phase favors the modulation of the SPR signal, by intensifying the resonance phenomenon. Studies by Liu *et al*.^7^ mention that the modification of the MoO_3_ surface, it will strongly be associated with more available free electrons near the surface, and they conclude that if the density of free electrons is increased; the SPR signal also increases. This behavior can be corroborated with the arising of a band and the red-shift which experiment this band. Likewise, Zhang *et al*.^18^ reported that the SPR effect is intensified when the free electrons concentrations increase on the surface. Zhang mention that the behavior is associated with the presence of oxygen vacancies and with a low crystalline structure. In addition, Linic *et al*.^6^ mention that one of the mechanisms of charge carriers transfer occurs on the semiconductor surface, under the assumption that the plasmonic nanoparticles and the semiconductor are in direct contact, allowing a rapid transfer of charge carriers. Therefore, the metal plasmonic nanoparticles essentially act by absorbing resonant photons and transfer the generate photo-electron, formed in the resonant excitation process, to the semiconductor.

Taking these studies into account, firstly it is considered what Linic *et al*. mentions as an assertion in our model. Moreover, considering that Liu and Zhang highlight the importance of the oxygen vacancies and the low crystallinity in the MoO_3_ structure and how these features are related to the SPR signal increase. So, it might be correlated that these characteristics are essential for increasing the SPR signal and enhanced the optical properties. According to the obtained results, the intensification of the SPR signal could be attributed to the presence of these three properties. In previous works has been demonstrated that the *β* – phase has a sub-stoichiometric amorphous structure associated with oxygen vacancies in the MoO_3_ matrix^30,44^ unlike the *α* – phase where its crystalline domain is bigger and available oxygen vacancies are less than *β* – phase^44^. The importance of oxygen vacancies to explain the increase in the SPR signal is of the utmost importance since these vacancies would function as electron traps, which could be absorbed the generated resonant electrons. Therefore, a larger number of photogenerated charge carriers, due to the resonance in a structure with higher oxygen vacancies density, the probability that these vacancies absorb these resonant electrons will be very high. Another aspect that supports the hypothesis that a higher free electrons density in this structure, higher intensity in the SPR signal, is the red-shift of the SPR dip, which is shown in the transmittance spectra of the *β* – phase, when it is compared with the *α* – phase^7^. In another way, the main contribution of the titanium nanoparticles, is weak on the MoO_3_ thin film compared with the gold nanoparticles. As expected no-resonance behavior is present in this case, so the changes in the transmittance signal are not significant. The simulation with a non-resonant particle is useful as a comparison between resonant and non-resonant particles and showing the potential of using noble metals as dopants in thin films for developing new devices. One thing that should be mentioned is that the concentration of the Ti nanoparticles just decrease a little the transmittance and have a shift on the maximum peak of transmittance. Meanwhile, if the thickness of the MoO_3_ increase, a bigger shift can be seen on the transmittance, which could be interpret as a shift due to the presence of a plasmonic resonance as Liu, *et. al*. reported^7^. The effect of the film thickness is not observed as well as in the case of embedded gold nanoparticles, due to the presence of the plasmonic nanoparticles which resonance signal is just after de peak of the transmittance, making this phenomenon not well remarkable as in the case of titanium particles, which are non-plasmonic particles.

## Conclusions

In this work, a new theoretical model was developed to characterize MoO_3_ thin films without doping and doping with resonant (Au) and non-resonant (Ti) nanoparticles, through the implementation of effective media theory. As can be seen, the applicability of the theoretical method, for the prediction of transmittance spectra can be corroborated by qualitative comparison with some experimental results. Among the analyzed parameters, the most important in the transmittance modulation is the nanoparticles concentration and the MoO_3_ phase. It was found that the higher the nanoparticles concentration (3.5%), the intensity of the SPR signal is higher. It is possible to modulate up to 0.55 on transmittance for the alpha phase and for the beta phase the modulation reaches a value of 0.85 on transmittance, so the beta phase of the MoO_3_, is the phase where the optical modulation is benefited. This result is associated with more oxygen vacancies density in the beta phase in which the generated photoelectrons in the resonance phenomenon, could be injected or trapped by the anionic vacancies present in the semiconductor. These results are important for the optical tuning devices design. Additionally, as a comparison, the changes in the transmittance spectrum are studied when the MoO_3_ is doped with Ti – NPs. Similar behavior is observed, where at a higher Ti nanoparticles concentration and for the *β* – phase, the band associated with MoO_3_ absorption suffers a red-shift. However, this cannot be compared with the changes presented in the Au – NPs case but it is interesting to observe that with the incorporation of Ti in MoO_3_ matrix it can be modulated the maximum absorption band. From the performed simulations, an interesting result has been found in MoO_3_ thin films. As in chromogenic materials, the SPR signal is also be favored when the MoO_3_ is in the *β* – phase. These results have many implications in technological applications, *e.g*., in the bio-sensing process, in energy efficiency or even more in harvesting electrons for clean energy generation.
